# Perceptions of the ‘good farmer’ and social licence to operate in Aotearoa New Zealand

**DOI:** 10.1080/03036758.2024.2351910

**Published:** 2024-05-26

**Authors:** Pamela L. Booth, Martin Espig, Nicholas Kirk, Jim Sinner, Hugh Campbell, Peter Edwards, Robyn Kannemeyer, Chloe Dear

**Affiliations:** a Manaaki Whenua – Landcare Research, Wellington, New Zealand; b M.E. Consulting, Christchurch, New Zealand; c Manaaki Whenua – Landcare Research, Lincoln, New Zealand; d Independent researcher, Nelson, New Zealand; e Department of Sociology, Gender Studies, and Criminology Dunedin, University of Otago, Dunedin, New Zealand; f Manaaki Whenua – Landcare Research, Auckland, New Zealand; g Quorum Sense, Canterbury, New Zealand

**Keywords:** Aotearoa New Zealand, social licence to operate, good farming, good farmer, social licence to farm, public perceptions

## Abstract

Over the past 50 years, farming practices and public perceptions of farming in Aotearoa New Zealand (AoNZ) have changed substantially. These changes raise questions over the future relationship between farming communities and the public. Recent research challenges the assumption that perceptions of farming differ significantly between farmers and the public. Using two recent surveys in AoNZ, we apply the social licence to operate concept using the ‘good farmer’ as a foundation. We find perceptions of ‘good farming’ practices held by the public and farmers are relatively aligned, but these groups may have different interpretations despite core messages aligning. Farmers were trusted as a source of information more than mainstream media by both groups. Increasing alignment of ‘good farmer’ perceptions between the groups can lead to an increase in perceived trustworthiness of farmers as an information source by the public. Our findings contribute to the emerging body of literature showing the perceived ‘rural/urban divide’ may not be as large as believed. These insights extend the literature by finding strong alignment of perceptions and nuanced interpretations of good farming practices by the public and farmers. Further investigation can contribute to constructive societal conversations about agriculture’s social licence in AoNZ.

## Introduction

Aotearoa New Zealand’s cultural identity and economy are intimately linked to farming and agriculture (White and Potts 2008; Hunt et al. 2013). However, over the past 50 years, farming practices as well as consumer and public perceptions of farming in Aotearoa New Zealand (AoNZ) have changed substantially (Fairweather and Campbell 2003; Kirk et al. 2022). Deregulation of the economy (including primary industries) in the 1980s led to shifts in farming practices that emphasised increasingly intensive productivist approaches (Haggerty et al. 2009). In recent decades public support for growth-focused economics and farming practices has declined (UMR Market Research 2008, 2017). These changes are reflected in evolving formal regulation (e.g. Campbell 2023), media messaging (e.g. Campbell 2023) and large-scale public surveys (e.g. Booth et al. 2022; Beban et al. 2023). These developments have raised deeper societal questions over the future of the country’s agricultural sector and, on the surface, appear to have affected the relationship between farming communities and the wider public.

A key concept to contextualise the social dynamics of farming is the ‘good farmer’ notion. Burton et al. (2020) describe ‘good farming’ as one of the most useful entry points to understanding farm practices and the way that farmers perceive their own role in environmental management and as contributors to rural communities (see also Burton 2004; Burton and Wilson 2006). Rural social scientists recognise that a powerful influence on farmer identity is the self-perception by farmers that their practices are ‘good’ and that they are seen as positive contributors to wider society and the national economy. This sense of being a ‘good farmer’ lies at the heart of what Burton (2004) saw as a crucial difference in the way that UK arable, and beef (Burton et al. 2020), farmers considered highly productivist and intensive farm practices compared to ‘post-productivist’ environmental management practices as legitimate and justified. Each set of practices rose out of the dramatically different way that farmers understood their practices as being ‘good’.

Burton (2004) provided the hook that enabled a group of social researchers examining environmental dynamics in farming to locate some of the drivers of change within the way that farmers perceived themselves, and their neighbours, as ‘good farmers’. Some researchers began to employ the concept to examine the way social pressures were applied to farmer behaviour (Isgren 2016; Burton et al. 2020, p. 5), to analyse how farmer identity interacted with government policies about the environment (Hansson et al. 2012) or in direct examination of how farmers understood and valued (or not) alternative farming practices (Sutherland and Darnhofer 2012).

As the concept evolved and was tested, farmers’ identities and sense of being ‘good farmers’ were linked in various ways to wider societal pressures, policy requirements, market demands and education (reviewed in Burton et al. 2020). This showed that different identities of good farming are not just determined by self-perceptions but also by their dynamic interplays with the views of the wider public and societal perceptions of what constitutes good farming practices.

These dynamics open up the space to ask questions about how good farming as a pillar of rural identity interacts with societal-level perceptions of the values and virtues of farming. Such evaluations are demonstrated by Dorner et al. (2023), who found that frontage of a farming operation on a busy road was positively associated with additional uptake of ‘good management practices’ that are easily recognisable by the public, even if other less recognisable ‘good management practices’ were also being implemented on-farm.

Given the importance of these connections in the good farming literature, it is concerning that there appears to be a strained relationship between farming communities and the AoNZ public, with two recent surveys showing that only 34% of farmers believe the public perceives on-farm environmental performance as ‘good’ to ‘very good’ (Stahlmann-Brown 2023), while only 40% of the public actually believing on-farm environmental performance is ‘good’ to ‘very good’ (Booth et al. 2022).

This paper examines farmer identity and ‘good farming’ by linking it to a parallel concept: social licence to operate (SLO). Social Licence to Operate exists as an unwritten contract (i.e. as the informal social expectations of the local community for a business), or as a structured regulatory and legal expectation of business practices (Moffat et al. 2016). This unwritten contract, one could argue, can involve a continuum of cultural norms and expectations of the relationship between the public and businesses or other entities. Just like cultural norms, the location of SLO along this continuum also changes as societal expectations, beliefs and values change.

Our research sought to understand and apply the concept of SLO to farming more uniformly, using the ‘good farmer’ concept as a proxy. The growing need for such an analysis is evident in anecdotal and media accounts that suggest the AoNZ public and regulators are disconnected from, and have skewed perceptions of, rural life and farm management practices. However, recent research appears to challenge the assumption that public and farmer perceptions of farming are all that different. For example, Diprose (2023) found that the majority of farmers see themselves as stewards of the land for future generations and use management practices that would be considered environmentally sustainable. Beban et al. (2023) also found that the urban public’s perceptions of farming are not as dissimilar to those of rural communities as the media portray. To better explore the anecdotal indication of a dichotomy between public and farmer perceptions of what constitutes ‘good farming’ and social licence to farm, this paper is guided by the following research questions:

- How well do the public’s perceptions of a ‘good farmer’ and farmers’ perceptions of a ‘good farmer’ align?
- Does increased alignment of perceptions increase the public’s confidence and trust in farmers and farming practices, and thereby its social licence?
- How do the public and farmers interpret the concept of being a good farmer?

We next describe the concept of social licence to operate and discuss its application in agriculture and farming. We then present the data collection and analysis methods before presenting and discussing our results.

## What is social licence to operate?

Compared to the ‘good farmer’ concept’s predominant focus on farmers’ individual and collective self-perceptions, SLO is a more relational concept describing inter-group expectations and engagements, often at a wider communal or societal level. While there is no single definition of, or set of characteristics for, SLO (Edwards and Trafford 2016) many researchers regard SLO in terms of an unwritten social contract on an often-local level between a community and business or industry (Thomson and Boutilier 2011; Parsons and Moffat 2014; Moffat et al. 2016). As such, SLO should not be considered in the same way as a legal licence, due to its intangible and dynamic nature. In the context of specific development projects, SLO can thus be regarded as the ‘ongoing and fluid level of acceptance by stakeholders, at multiple levels, which may be revoked at any stage of the project lifecycle based on changes in perceptions, and reflective of the relationships between a company and its external stakeholders’ (Mercer-Mapstone et al. 2017, p. 347). If considered as this complex value-based concept and informal licence, SLO must not only be earned but also maintained through continuous (re)negotiations with diverse actor groups. This occurs across a multitude of sociocultural engagements that go beyond formal ‘stakeholder consultation’ or ‘community engagement’ programmes.

The concept first emerged in the mid-1990s to describe proactive environmental policies implemented by the American forest and paper industry aimed at building public trust in the industry (Moore 1996; Cooney 2017). Since then, SLO discourses have prominently featured in different extractive industries and national contexts, most notably in the international mining sector where it is linked to other concepts such as corporate social responsibility (Dumbrell et al. 2020), human rights due diligence, and free, prior and informed consent (FPIC), and gained popularity as a tool for managing community relations or ‘social risks’ (Lehtonen et al. 2022). Among those, SLO constitutes the most basic (i.e. ‘weakest’), yet most publicly used, social performance concept. The SLO’s conceptual weaknesses, such as its binary implications of either ‘having’ or ‘lacking’ social acceptance and often tokenistic implementation across the mining sector have been substantially critiqued by some researchers (e.g. Owen 2016; Meesters and Behagel 2017; Owen and Kemp 2017). While covering these critiques in detail is beyond the scope of this article, it is worth noting that SLO has commonly been employed across the mining sector rather superficially and often with a primary focus on managing business ‘risks’ or ‘reputation’ by ensuring a minimum of community resistance (Owen and Kemp 2017). This interpretation of SLO is at odds with the more relational understanding outlined above and how we operationalise the concept for this study.

Notwithstanding these and other criticisms, SLO has featured in a range of other sectors, with some observers contending that any organisation might need a social licence (Morrison 2014). Contexts where the concept has been employed to describe industry-community relationships and instances of potentially contested social acceptability include aquaculture and marine management (Cullen-Knox et al. 2017; Kelly et al. 2017, 2019; Baines and Edwards 2018; Sinner et al. 2020; Edwards et al. 2021), forestry (Dare et al. 2014; Edwards et al. 2016, 2019a), energy industries (Hall et al. 2015; Shaffer et al. 2016; Mabele et al. 2023), conservation (Oakes et al. 2015; Kendal and Ford 2018), hydroelectric power (Jijelava and Vanclay 2018), nuclear waste management (Lehtonen et al. 2022), invasive species control (Kirk et al. 2019), and livestock farming (Coleman 2017). Apart from noteworthy exceptions (e.g. Martin and Williams 2011), questions concerning the social licence of farmers and the agricultural sector have so far received limited attention in international scholarship, although the notion has been promoted in industry discourses and grey literature where agricultural land uses, such as intensive dairying, are under growing public scrutiny (e.g. Castka et al. 2023).

Trust, trustworthiness, legitimacy, and credibility are recognised as key concepts associated with SLO (Boutilier and Thomson 2011; Moffat and Zhang 2014; Jijelava and Vanclay 2018; Dumbrell et al. 2020; Lehtonen et al. 2022). Where interpersonal and institutional trust are fostered between institutions and actors (e.g. between farmers and government agencies), collective action is more likely to be facilitated. Moffat and Zhang (2014) suggest that SLO could be used to build consensus when there are diverse perspectives on a topic of contention, particularly in terms of building trust, trustworthiness, and fairness with communities of interest. Lehtonen et al. (2022) suggest that SLO scholarship needs to embrace the multiple dimensions of trust, interpersonal (social), institutional, and ideological, together with the dynamics that exist between trust, mistrust, and distrust. Trust is an inherently complex concept (Welter 2012) and we here only examine one of the key elements of trust and trustworthiness in relation to the agricultural sector’s ‘social licence to farm’: the public’s assessment of trustworthiness of sources of information about farming.

The SLO concept is used sporadically by practitioners in AoNZ; this includes critical reflections on how it relates to the values and experiences of Māori (the country’s indigenous people) in resource developments (Ruckstuhl et al. 2014; Edwards and Trafford 2016; Edwards et al. 2019b). However, few targeted research efforts have explored social licence-related challenges emerging for the country’s agricultural sector. This sector remains a critical source of revenue and is central to cultural identity in rural regions (e.g. Beban et al. 2023). At the same time, agricultural land uses play a crucial role in a range of pressing challenges for the nation’s terrestrial, freshwater, and marine ecosystems, such as reducing greenhouse gas emissions and nutrient losses from livestock farming (McDowell 2021). This article engages with these changing community sentiments and, in response, evolving (self-) perceptions of the ‘good farmer’ among agricultural actor groups. It also addresses, more broadly, comparably limited engagements with the SLO concept in the broader agricultural sector within international scholarship.

## Data and methods

### Data collection

The data were collected through two online surveys in May and June 2023 across AoNZ with primary producers^1^ (i.e. farmers) and the public. The first survey was designed to explore the general population’s perceptions of what it means to be a ‘good farmer’ (public survey). The public survey consisted of 28 quantitative and qualitative questions that were informed by the ‘good farmer’ literature, Open Farms Day surveys,^2^ and farmer interviews.^3^ Respondents were recruited through a commercially purchased panel representative of the AoNZ public in terms of age, gender, ethnicity and region. Respondents who completed the survey received an unspecified incentive from the commercial panel provider. The second survey was of farmers’ perceptions of ‘good farming’ (farmer survey). The farmer survey consisted of 31 quantitative and qualitative questions that were informed by the ‘good farmer’ literature and farmer interviews. Respondents were recruited through the researchers’ existing networks and their industry partners, with an incentive of a $5 donation to a charity of choice offered for completion.^4^ Both surveys were enumerated online using the online survey platform Qualtrics and were programmed using adaptive logic that tailored questions and survey flow to each respondent. All questions were optional in both surveys.

The public survey received a total of 1,217 responses of which 48.5% were male, 66% of New Zealand European descent, 31.6% resided in Auckland, and 55.9% were between the ages of 20 and 39-years. Sixty-four percent of public survey respondents had interacted with a farmer, 62.5% lived within 15 km of a working farm, 62% lived in a city and 10% lived in a rural area. The farmer survey received 25 responses of which 43.5% were male, 80% of New Zealand European descent, 16% Māori and 60.9% were at least 50-years of age. Farmer survey respondents were predominately from the Auckland (20%), Wellington (20%), Canterbury (12%) and Southland (12%) regions. Those who identified as Māori were over-represented in the public survey sample (+11%pts.) compared to the 2018 New Zealand general population census^5^ while, in the farmer survey, those who identify as female (+24.6%pt.), those in Auckland (+9.7%pts.) and those in Wellington (+14.9%pts.) were over-represented compared to the 2021 Survey of Rural Decision Makers (Stahlmann-Brown 2023).^6^

There were some minor differences in wording between the public and farmer surveys. For example, ‘good farmer’ was used in the public survey while ‘good farming’ was used in the farmer survey. This difference was a result of unprompted comments during the piloting stage of the farmer survey. Some farmers felt that the term ‘good farmer’ conferred a judgement on the individual, whereas the term ‘good farming’ conferred a judgement on farming practices. We did not explore the impact of this semantic difference but recognise that perceptions are influenced by different lived experiences and that some farmers and/or the public may be sensitive to one phrasing or the other. The testing of the public survey did not identify any issue with the term ‘good farmer’, but pilot respondents were also not prompted to consider an alternative phrasing. The public survey was already fully deployed prior to the pilot testing of the farmer survey. We use the term ‘good farmer’ in this paper to refer to both ‘good farmer’ and ‘good farming’ for consistency and to align with the ‘good farmer’ literature phraseology.

### Methods for research question 1

Respondents to the public and farmer surveys were presented with a list of 20 characteristics associated with being a ‘good farmer’ derived from the literature (e.g. Burton and Wilson 2006) and farmer interviews. Respondents to the public survey first chose the characteristics that ‘matter most’ to them in terms of ‘what makes a good farmer’. They were then asked to score the level of importance of up to 10 of their chosen characteristics^7^ on a seven-point Likert scale from 1 equalling ‘Not at all important’ to 7 equalling ‘Extremely important’.^8^ Respondents to the farmer survey were asked to score the level of importance for each of the 20 characteristics on a four-point Likert scale from 1 indicating ‘Not important’ to 4 indicating ‘High importance’.^9^ Respondents to the public survey chose a median of 10 characteristics that ‘matter most’ to them and provided an importance score for a median of seven of those characteristics while respondents to the farmer survey provided an importance score for a median of 20 characteristics.^10^

Public and farmers’ perceptions of ‘good farmer’ characteristics were compared in two ways. The first was a descriptive comparison of the proportion of the public that chose a given characteristic as being associated with being a ‘good farmer’ and the proportion of farmers that scored that characteristic as having ‘High importance’. The second analysis rescaled the farmers’ importance score to a seven-point scale to enable the use of multi-linear regression to understand the significance and magnitude of difference between the public’s and farmers’ scores.^11^ The functional form of the multi-linear regression model is shown in Equation (1) where $GF_{\mathrm{ij}}^{k}$ represents the observable latent level of importance that individual i gave characteristic j in survey type k.

(1) $$GF_{\mathrm{ij}}^{k}=\;{\text{β}}_{0}+{\text{β}}_{1}\mathrm{Publi}c_{i}+x_{i}^{\prime }\text{β}+\epsilon_{i}\;\mathrm{where}\;\;i=1,\;\ldots ,n_{k};\;j=1,\;\ldots ,\;20;\;k=\{\begin{array}{c} \;p\;\mathrm{if}\;\mathrm{public}\\ \;f\;\mathrm{if}\;\mathrm{farmer} \end{array}\;\;\;$$

$GF_{\mathrm{ij}}^{p}\in [1,\;2,\;3,\;4,\;5,\;6,\;7]$ represents a response to the public survey and $GF_{\mathrm{ij}}^{f}\in [1,\;\;3,\;\;5,\;\;7]$ a response to the farmer survey. The independent variable $\mathrm{Publi}c_{i}$ was defined as 1 if individual i responded to the public survey and 0 if individual i responded to the farmer survey. ${\text{β}}_{1}$ is the coefficient of interest and represents the average difference in importance score that the public gave characteristic j compared to farmers for that same characteristic. A vector $x_{i}^{\prime }$ for age, gender and ethnicity was included as a control, with robust errors $\epsilon_{i}$. Public survey and farmer survey respondents who did not provide an importance score for that characteristic were excluded from the analysis for that characteristic.

### Methods for research question 2

We created a binary variable $\mathrm{Alig}n_{\mathrm{ij}}$, shown in Equation (2), for each of the j=1,…,20 ‘good farmer’ characteristics.

(2) $$\begin{array}{rl} & \mathrm{Alig}n_{\mathrm{ij}}=\\ & \;\left\{ \begin{array}{c} 1\;\mathrm{if}\;\mathrm{Pr}\;\left(GF_{\bar{i}j}^{f}-z*\frac{s(GF_{\bar{i}j}^{f})}{\sqrt{n^{f}}}\leq \;GF_{\mathrm{ij}}^{p}\leq GF_{\bar{i}j}^{f}+z*\frac{s(GF_{\bar{i}j}^{f})}{\sqrt{n^{f}}}\right)=0.99\;\mathrm{where}\;z∼N(0,\;1)\;\\ 0\;\mathrm{if}\;\mathrm{else}\;\; \end{array}\right. \end{array}$$

For each characteristic j we averaged importance scores across farmer respondents $\left(GF_{\bar{i}j}^{f}\right)$ then we compared this average to each public survey respondent’s score $GF_{\mathrm{ij}}^{p}$. If the public survey respondent’s score was within the *99%* confidence interval of the farmer average, then that public survey respondent was considered in alignment with farmers for that attribute and $\mathrm{Alig}n_{\mathrm{ij}}=1$. If the public survey respondent’s score was outside of the *99%* confidence interval, then they were considered unaligned and $\mathrm{Alig}n_{\mathrm{ij}}=0$. Public survey and farmer survey respondents who did not provide an importance score for that characteristic were excluded from the analysis for that characteristic. Each public survey respondent could align with farmers with up to 10 out of the possible 20 good farmer characteristics due to the constraints in the public survey, as outlined in Section 3.2.

Trust in farmers was measured using perceived trustworthiness of farmers as a source of information. Respondents to the public and farmer surveys were asked to score the level of trustworthiness of 15 different sources of information on a seven-point Likert scale from 1 equalling ‘Completely untrustworthy’ to 7 equalling ‘Completely trustworthy’. Similarly to research question 1, we use a multi-linear regression to understand the significance and magnitude of a change in level of trustworthiness of farmers as a source of information, using the observable variable ${\mathrm{Trust}}_{\mathrm{iF}}^{k}\in [1,\;2,\;3,\;4,\;5,\;6,\;7]$ for the latent variable ${{\mathrm{Trust}}_{\mathrm{iF}}^{k}}^{*}$, held by the public (k=p) as the number of aligned good farming characteristics increase (Equation 3).

(3) $${\;\mathrm{Trust}}_{\mathrm{iF}}^{p}=\;{\text{β}}_{0}+{\text{β}}_{1}\mathrm{AlignCoun}t_{i}+x_{i}^{\prime }\text{β}+\epsilon_{i}\;\;\mathrm{where}\;i=1,\;\ldots n_{p};\;\mathrm{AlignCoun}t_{i}=\overset{20}{\underset{\;j=1}{\sum }}\mathrm{Alig}n_{\mathrm{ij}}$$

The independent variable of interest $\mathrm{AlignCoun}t_{i}$ takes on values 0 through 10 depending on the number of ‘good farmer’ characteristic perceptions that public respondent i holds that align with farmers’ perceptions. ${\text{β}}_{1}$ is a vector of coefficients of interest that represent the change in perceived trustworthiness of farmers as a source of information held by the public as the number aligned characteristics increase from 0 to 10. A vector $x_{i}^{\prime }$ of age, gender and ethnicity are included as controls with robust errors $\epsilon_{i}$.

### Methods for research question 3

We used content analysis to examine the qualitative survey responses (Stemler 2015). This approach allowed us to capture and classify insights from the research question: ‘How do the public and farmers interpret the concept of being a good farmer’. The content analysis was carried out in MS Excel initially by one researcher and the results were cross-checked independently by two other researchers. All three researchers discussed and refined the content.

## Results

### How well do the public’s and farmers’ perceptions of a ‘good farmer’ and farming align?

**Figure 1.**
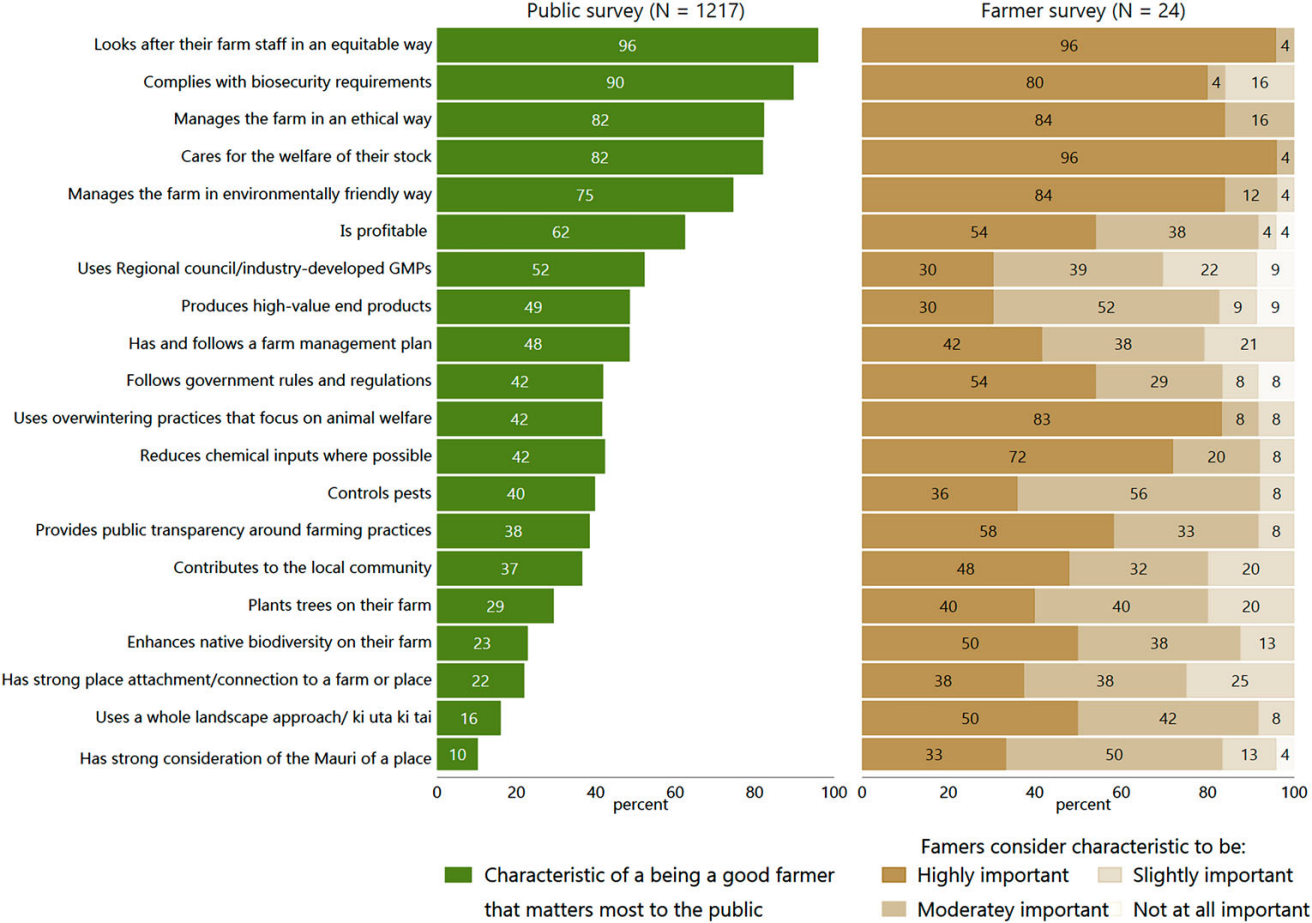
Proportion of respondents to the public survey who identified ‘good farmer’ characteristics as one that mattered most to them (left panel) versus the perceived importance of ‘good farmer’ characteristics by respondents to the farmer survey (right panel).

Nearly all respondents to the public and farmer surveys thought that ‘Looks after their farm staff in an equitable way’ was a ‘good farmer’ characteristic that ‘matters most’ to them/was ‘highly important’ (thereafter highly important; Figure 1). The majority of respondents to the public and farmer surveys also thought that ‘Complies with biosecurity requirements’, ‘Manages farm in an ethical way’, ‘Cares for the welfare of their stock’, ‘Manages the farm in an environmentally friendly way, and ‘Is profitable’ were ‘good farmer’ characteristics that were highly important. A similar proportion of respondents to the public and farmer surveys thought that ‘Has and follows farm management plan’ and ‘Controls pests’, were ‘good farmer’ characteristics that were highly important. More public respondents than farmers thought that ‘Uses Regional Council/industry-developed Good Management Practices (GMPs)’ and ‘Produces high-value end products’ were ‘good farmer’ characteristics that were highly important. More farmers than public respondents thought that the remaining 10 ‘good farmer’ characteristics were highly important.

**Figure 2.**
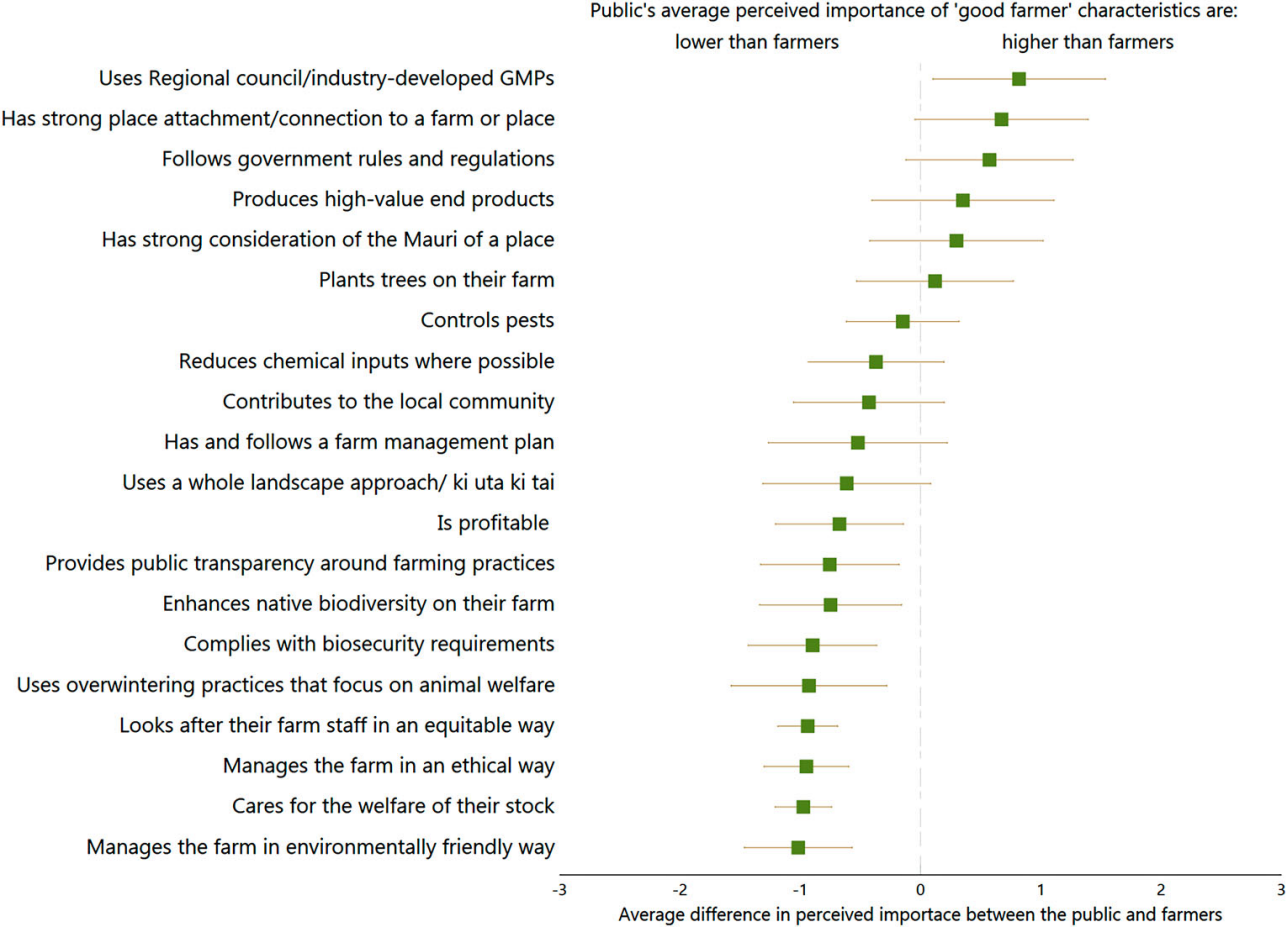
Average estimated difference between the public’s perceived importance of ‘good farmer’ characteristics and farmer’s perceived importance of ‘good farmer’ characteristics. Notes: Lines represent the 95% confidence interval of the estimated average difference whereby if the line does not cross ‘0’ then the difference is statistically different at the 5% level. Estimates and 95% confidence intervals are from the multi-linear regression model discussed in section 3.2. Full regression results are reported in Tables SM1a – c in the Supplementary Material.

For the following 10 characteristics, the difference between average public and farmer importance scores were not statistically significantly different at the 5% significance level (Figure 2):

- Has strong place attachment/connection to a farm or place, 95%CI[−0.06,1.38]
- Follows government rules and regulations, 95%CI[−0.09,1.29]
- Produces high-value end products, 95%CI[−0.34,1.19]
- Has strong consideration of the mauri of a place, 95%CI[−0.29,1.21]
- Plants trees on their farm, 95%CI[−0.48,0.87]
- Controls pests, 95%CI[−0.54,0.40]
- Reduces chemical inputs where possible, 95%CI[−0.87,0.25]
- Contributes to the local community, 95%CI[−1.03,0.23]
- Has and follows a farm management plan, 95%CI[−0.90,0.74]
- Uses a whole landscape approach/ ki uta ki tai, 95%CI[−1.30,0.17]

The regression results confirmed that the public thought ‘Uses Regional Council/industry-developed GMPs’ was more important to being a ‘good farmer’ than farmers and that the remaining 9 out of 20 characteristics were less important to being a ‘good farmer’ than to farmers.

### Does increased alignment of ‘good farming’ perceptions increase trust in farmers?

Alignment of the perceived importance of the 20 ‘good farmer’ characteristics varied across those characteristics: from 62.6% of the public who agreed with farmers on the importance of ‘producing high-value end products’ to 19.1% of the public who agree with farmers on the importance of ‘managing the farm in an environmentally friendly way’ (Table 1). The majority of public respondents agreed with farmers on the level of importance for 7 out of 20 characteristics, including characteristics emphasising animal/stock welfare (e.g. ‘Uses overwintering practices that focus on animal welfare’), biosecurity (e.g. ‘Controls pests’), environmentalism (e.g. ‘Plants trees on their farm’), and local place attachment (e.g. ‘Has strong place attachment/connection to a farm or place’).

**Table 1.** Proportion (%) of public respondents whose perceptions of importance of ‘good farmer’ characteristics align with farmers.

‘Good farmer’ characteristic	Proportion (%)^1^ of public whose perceptions of importance align with farmers	Number of observations (*n*)
Produces high-value end products	62.6	270
Controls pests	61.3	576
Has strong place attachment/connection to a farm or place	56.8	673
Contributes to the local community	56.8	190
Plants trees on their farm	56.6	366
Complies with biosecurity requirements	56.0	455
Has strong consideration of the Mauri of a place	51.1	180
Uses overwintering practices that focus on animal welfare	50.8	122
Has and follows a farm management plan	45.5	110
Follows government rules/regulations	41.2	758
Reduces chemical inputs where possible	37.3	474
Uses Regional Council or industry-developed GMPs	34.0	497
Is profitable	33.9	245
Looks after their farm staff in an equitable way	31.1	700
Cares for the welfare of their stock	30.3	459
Enhances native biodiversity on their farm	28.7	258
Manages the farm in an ethical way	26.8	426
Provides public transparency around farming practices	24.3	437
Uses a whole landscape approach/ ki uta ki tai	20.9	148
Manages the farm in an environmentally friendly way	19.1	487

^1^ Proportions are among public respondents who indicated level of importance for that characteristic.

Public respondents whose perceptions aligned with farmers for 2–5 characteristics tended to be older, more likely to be female, and more likely to identify as a New Zealander of European descent than respondents whose perceptions of characteristic importance did not align with farmers. Respondents whose perceptions aligned with farmers for 3–6 characteristics were also less likely to identify as Māori (Table SM2 in Supplementary Material).

**Figure 3.**
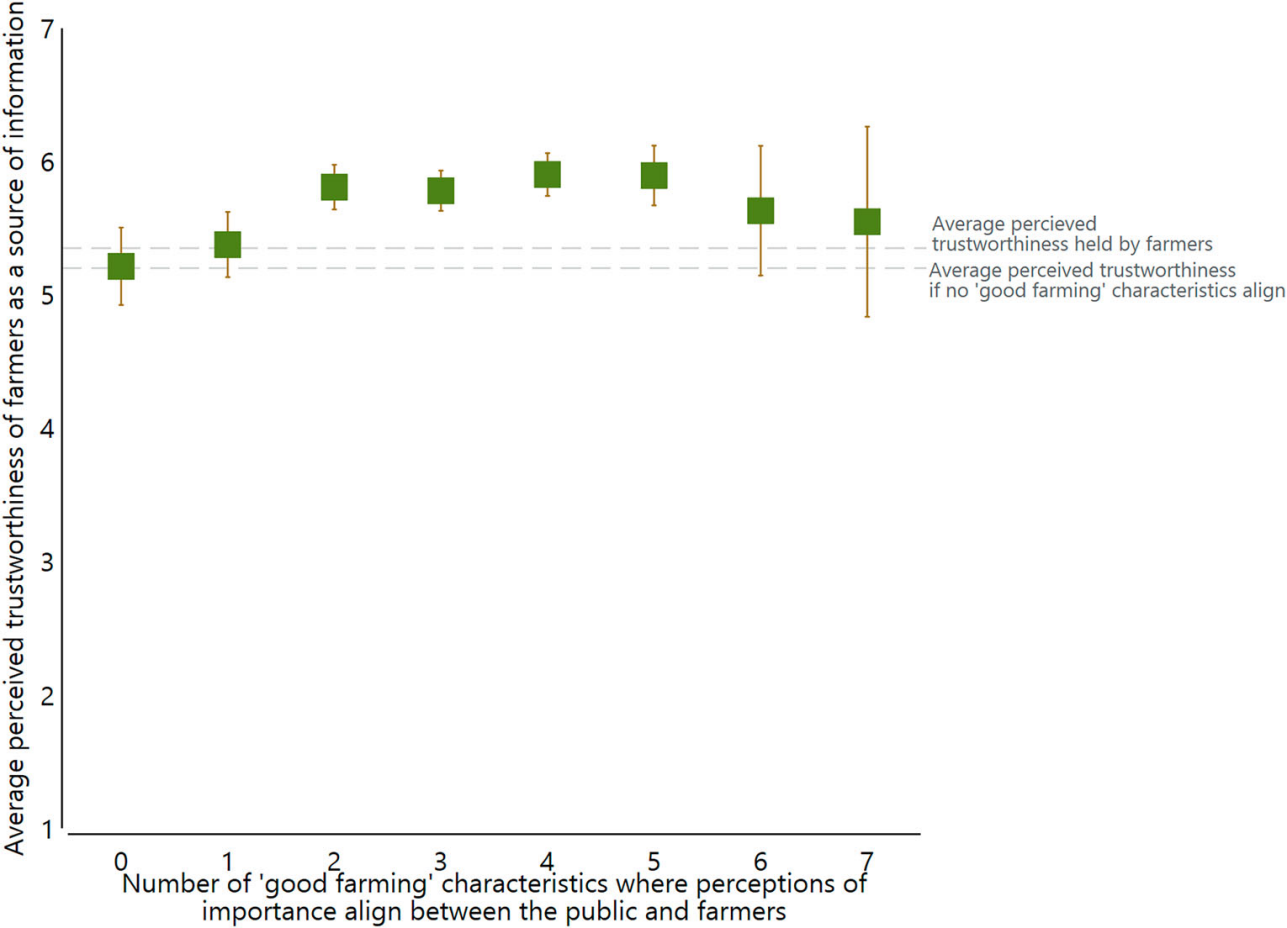
Average public’s perceived trustworthiness (scale 1–7) of farmers as a source of information by number of ‘good farmer’ characteristics that align between the public and farmers. Notes: Lines represent 95% confidence interval of the estimated average. Estimates and 95% confidence intervals are from the multilinear regression model discussed in section 3.3. Full regression results are reported in Table SM3 in the Supplementary Material.

As the number of ‘good farmer’ characteristics that align increased, the average perceived trustworthiness of farmers as a source of information held by the public also increased (Figure 3). The perceptions of trustworthiness held by the public whose perceptions align with farmers for one characteristic were similar to the average perceptions of trustworthiness held by farmers. However, as the number of characteristics that align increased, the public’s perceived trustworthiness of farmers exceeded farmers’ perceived trustworthiness of themselves. These results held when variables for familiarity with the agricultural sector and residence in an urban/rural area were included (Table SM3 in Supplementary Material). A follow-up analysis restricting the model sample to only those whose perceptions of being a ‘good farmer’ aligned with farmers for 1–10 characteristics confirmed these results (Table SM4 in Supplementary Material).

### How do the public and farmers interpret the concept of being a good farmer?

In analysing the qualitative responses to the surveys, the major challenge in comparing farmer and public perspectives of what constitutes a ‘good farmer’ is the significantly higher number of responses we received from the public. This resulted in the public responses being more diverse than the farmer ones. Despite the different numbers of responses across the two surveys, noteworthy insights can be drawn from a robust comparative analysis of how the public and farmers interpret the concept of being a good farmer.

#### How do perceptions and interpretations of being a ‘good farmer’ align between the public and farmers?

Analysis of the qualitative responses identified four characteristics that were found in both the farmer and public responses as to what makes a ‘good farmer’: ‘Animal welfare and care for stock’, ‘Environmental sustainability’, ‘Meeting consumer expectations and creating good healthy food’, and ‘Enhancing biodiversity’.

##### Animal welfare and care for stock

There is some evidence to suggest farmers and the public interpret animal welfare and care of stock differently. For example, when asked to list any other criteria that might be used to judge ‘good farming’, one farmer argued that being ‘sympathetic to consumer expectation regarding animal welfare and environmental issues’ was one criterion (Farmer 1). Being sensitive to the public and consumers about animal welfare is another aspect, but it seems that some public survey respondents thought that being a ‘good farmer’ requires a significant shift in many current land use practices. For example, one public respondent stated that being a ‘good farmer’ meant being ‘willing to transition to plant-based crops given how cruel, and damaging to climate change, animal agriculture is’ (Public respondent 1). This respondent was clear about the change they thought needed to occur to be a ‘good farmer’ and to obtain social licence, but in most cases public responses regarding animal welfare were vague about what this actually meant in practice.

##### Environmental sustainability

Public survey respondents articulated sustainability as being ‘eco-friendly’, reducing ‘carbon footprints’ or ‘environmental footprints’ more generally, ‘caring for the environment’, and caring for ‘their farm’ or ‘land’ more generally. In contrast, farmers discussed ‘holistic farming’ and noted how environmental sustainability was often linked to other issues, with one farmer responding as below when asked what characteristics they might use to judge being a ‘good farmer’:

Farming to produce good food and produce, in a good culture for people and minimising impacts on the environment and enhancing biodiversity. (Farmer 2).

##### Meeting consumer expectations and creating good healthy food

Public survey respondents expressed a variety of interpretations of how ‘Meeting consumer expectations and creating good healthy food’ is a ‘good farmer’ characteristic. These interpretations were articulated as farmers ‘having quality control for their products’, ‘exhibiting food safety practices and hygiene’, and ‘meeting food grade certification standards’. For some public respondents, obtaining or maintaining a social licence meant that ‘All farming should follow all rules to keep our animals and food healthy and safe for people to eat’ (Public respondent 2).

Farmers also expressed a desire for agricultural practices and land use systems that ‘minimise chemical inputs’ and ‘enable growing organic produce’, similar to the public’s interpretations of good farming. To emphasis this, when asked what first comes to mind when thinking of farming, one farmer responded:

The insistence of NZ farmers to pursue a chemical-based approach will eventually shut them out from international markets where the consumer wants organic food responsibly produced. (Farmer 3)

This shows that perceptions of being a ‘good farmer’ between the public and farmers may not necessarily be as polarised as portrayed by some media or perceived by some in the agricultural sector (e.g. Campbell 2023).

#### Enhancing biodiversity

As noted in the ‘Environmental sustainability’ section above, one farmer spoke of producing good food while also enhancing biodiversity. This characteristic was also articulated by many of the public respondents. For example, when asked to list additional characteristics of being a ‘good farmer’ one public respondent noted that, ‘Good farmers should minimise chemical inputs and enhance the farm’s native biodiversity’ (Public respondent 3).

Similar to the animal welfare theme, protecting and enhancing native biodiversity was frequently noted by public respondents as an important characteristic, but most did not elaborate on exactly what this entails in practice. For the respondent mentioned above, ‘enhancing biodiversity’ is linked to a shift away from chemical inputs, while other public survey respondents articulated the desire to shift away from chemical inputs without the connection to biodiversity.

#### Where are the differences and similarities of perception of ‘good farming’?

Various characteristics of being a ‘good farmer’ were commonly mentioned by the public but not articulated by farmers. Some of these include treating staff well, keeping fences intact, minimising the use of chemical inputs, ensuring they meet environmental requirements and legislation, and that they produce a good yield during harvest. It is difficult to confirm if farmers did not list these because they interpret the characteristics of a ‘good farmer’ and ‘good farming’ differently, or if this is because of the relatively smaller sample size of farmer response in comparison with the public.

One characteristic that farmers frequently expressed – which was not expressed by the public – was the social acceptability of farming itself. According to farmer respondents, a ‘good farmer’ was one who gained ‘social acceptability’ for their practices. Simultaneously, farmers expressed a belief that many members of the public do not understand what farmers do, and that this lack of understanding could affect public perception of the acceptability of farming. One farmer articulated the following response when asked if being a ‘good farmer’ helps them maintain or gain social licence:

My definition of a ‘good farmer’ may not align with that of such members of the ‘public’ as SAFE [Save Animals from Exploitation], Greenpeace, the vegan lobby etc. so even being a ‘good farmer’ by my standard can still be subject to social media criticism by the mere fact that we have animals that are not free to roam wild, no matter how we love and care for them. (Farmer 4)

This quote highlights that some farmers recognise there is a spectrum of opinions about their farming practices, and on one side of the spectrum, represented by the groups they listed, members of the public may fundamentally never accept certain practices, like livestock farming, as being ‘good’.

In response to the first part of the research question, there were at least four characteristics of a ‘good farmer’ where the public and farmers agreed. Due to the gap in sample size between both groups, public responses were more varied and broader, making it hard to answer definitively whether perceptions align. However, as Farmer 4’s quotation highlights, farmers recognise that certain members of the public’s perception of ‘good farming’ will probably not align with their own, due to fundamental differences of opinion.

## Discussion

We found that the public and farmers surveyed in this research have similar perceptions of the term ‘good farmer’ that in general related to farmers’ practices on their land; this has also been identified as a key theme/finding by other researchers (Beban et al. 2023; Diprose 2023). Perceptions of ‘good farmer’ characteristics align between the public and farmers when those characteristics fall into the broad categories of ‘On-farm animal welfare’, ‘Supporting biodiversity on-farm and farming in an environmentally friendly way’ and taking a ‘Whole landscape approach and connection with the land that considers the mauri (life force) of the land’. These findings resonate with those of Beban et al. (2023) who compared perceptions across the urban-rural ‘divide’ and found that the environmental impact of farming was a common concern across urban, rural and urban/rural respondents. Yet, supposed dissonances between farmers and parts of the wider AoNZ public increasingly feature in media accounts and on social media platforms (e.g. Groundswell NZ^12^), with a general feeling among some farmers that the urban public and regulatory agencies believe farmers are solely responsible for environmental declines (e.g. Hall et al. 1999). This prompts questions around why such disconnects between broadly aligned ‘good farmer’ perceptions and the agricultural sector’s perceptions of their SLO may exist.

Our study suggests that possible sources of disconnect lie in different interpretations of farming practices and differing perceptions of the natural environment. For example, we found that the public and farmers may interpret ‘sustainable farming’ differently even if the core ideas expressed are similar. As research in similar settings has shown, these findings could be explained by different personal characteristics, values, beliefs and culture (Hornsey et al. 2016; Daryanto and Song 2021), different personal experiences with the environment, and mental anchoring to past information or experiences (Kaur et al. 2004; Verbrugge and van den Born 2018). Further exploration of these differing interpretations and the potential influence of values, lived experiences and behavioural anchoring is needed.

Following on from this finding, our results have noteworthy implications for societal discussions about the sector’s social licence to farm. As an unwritten social contract, gaining a SLO generally requires building trust, trustworthiness, and legitimacy across different communities of interest (Boutilier and Thomson 2011; Moffat and Zhang 2014; Jijelava and Vanclay 2018; Lehtonen et al. 2022), which necessitates at least some degree of shared purpose and interests. In the case of the agricultural sector’s SLO, an understanding of what constitutes good farming practices and desired environmental, social, and economic outcomes forms the basis for such shared interests. Where these understandings (partially) diverge, as in the characteristics our study highlighted, different communities of interest may, more or less tacitly, seek dissimilar or even conflicting outcomes from agricultural practices; and this will have relevant negative implications for a shared sense of their social acceptability. Our findings help to identify key areas of potential misalignment in support of more explicit and transparent societal discussions, which are critical for building more trusted relationships across the spectrum of rural and urban actors to enhance the agricultural sector’s social licence to farm.

A disconnect between perceptions of farming and SLO may also occur because of variations in information or media upon which different actors base their views. For example, in a recent technical report on SLO and the rural-urban divide, Beban et al. (2023) found that the media was a significant influence on people’s perceptions of farming, with some members of the urban public perceiving the agricultural sector as engaging in public relations spin while many/some members of the rural public considered that the mainstream media (e.g. national newspapers and TV programmes) was only focusing on, or sensationalising, supposedly negative aspects of farming. However, our findings that farmers were trusted as a source of information more than mainstream media (e.g. TV news, radio news, TV programming) by both the public and farmers suggests a more nuanced story relating the influence of media on perceptions of farming.

International research shows that trust is a significant factor in building social licence (Edwards et al. 2019b; Lehtonen et al. 2022). Exploring one dimension of trust, we found that higher alignment of what it means to be a ‘good farmer’ between the public and farmers and perceived trustworthiness of farmers as a source of information held by the public is positively linked. This adds an important insight to the existing scholarship on trust and inter-group dynamics in the context of societal discourse over the agricultural sector’s SLO. However, further research will be required to determine underlying causal relationships for our finding and to see whether similar dynamics can be observed regarding SLO negotiations in other sectors.

Notwithstanding the need for more targeted research into these inter-group dynamics, our results demonstrate that farmers and the public are relatively aligned on what ‘good farming’ means. This alignment can provide an opportunity to ‘break the ice’, renew conversations, and build trustworthy relationships. These steps are important components of building, maintaining, and re-building social licence, as necessary, through the building of relationships between farmers and the public. This is where different spaces and places may provide opportunities for farmers and the public to meet and build relationships, leading to the creation of trust and providing opportunities for constructive debates over what constitutes good farming practices. Given the fluidity and dynamics involved in the ongoing societal SLO discourse (e.g. Thomson and Boutilier 2011; Mercer-Mapstone et al. 2017), fostering and maintaining strong relationships between rural and urban members of the public in AoNZ will be crucial for strengthening the agricultural sector’s social licence to farm.

## Conclusions

Mainstream messaging and perceptions that the public and the farming community have widelydivergent opinions on what constitutes a ‘good farmer’ risk oversimplifying a diverse and complex spectrum of nuanced perceptions. Findings from our study support a growing body of literature that shows that the perceived ‘rural/urban divide’ is not as large as believed by some and extends the literature by finding that there exists both strong alignment of perceptions and nuanced interpretations of good farming practices by the public and farmers alike.

Perceptions, expectations and opinions also change over time (e.g. UMR Market Research 2008, 2017; Booth et al. 2022) which has implications for the agricultural sector’s SLO. Continuing to understand the dynamic relationship between the public and the agricultural sector through varying lenses is important. While we used the ‘good farmer’/’good farming’ concept, our study identified other potential areas of exploration linked with the SLO concept. These areas included perceived differences between an individual positively contributing to society by being a ‘good farmer’ and ‘good farming’ practices across the agricultural sector that positively contributing to society, interpretations of specific farming practices differing across different segments of a community and economy, and determinants of trust between the public and agricultural sector. Given the sociocultural and economic importance of the agricultural sector in AoNZ, investigating these areas further could contribute to the constructive societal conversation about the sector’s ‘social licence to farm’.

## Supplementary Material

Supplemental material

## Notes

1 Primary producers include those involved in sheep and beef farming, dairying, deer, pig, poultry and other livestock farming, horticulture, arable farming, foresters and/or beekeepers either for commercial or lifestyle purposes.

2
https://ourlandandwater.nz/outputs/sustainability-through-farmer-citizen-connection/

3 Famers at nine farming operations in two regions of AoNZ that represent a mixture of farming sectors, farm types and sizes, and ownership structures were interviewed in December 2022. Results from these interviews are not reported in this study but informed the surveys.

4 Respondents could choose from the New Zealand Red Cross (https://www.redcross.org.nz), New Zealand Rural Support Trust (https://www.rural-support.org.nz), Rural Women New Zealand (https://ruralwomennz.nz), and Mental Health Foundation (https://mentalhealth.org.nz).

5 The 2018 New Zealand Census is the most recent population survey available. As of the writing of this article results to the 2023 Census have yet to be released by Statistics NZ.

6 Demographic information for farm owners is not available from Statistics NZ. The Survey of Rural Decisions Makers is considered representative of the agricultural sector across industry and region compared with the New Zealand Business Demography Statistics for the agricultural, forestry and fishing industry (Australian and New Zealand Standard Industrial Classification 06 units A01 to A05 and therefore considered a reliable source of demographic information on farmers in New Zealand).

7 Randomly chosen from the complete list of characteristics that ‘matter most’ to that respondent.

8 The public were asked to rank up to 10 characteristics in order to minimise survey fatigue, as they may not have been as invested in the topic as were farmers.

9 Likert scale differences between the surveys was due to researcher oversight in not ensuring that they were identical.

10 The distribution of number of characteristics chosen by the public was highly concentrated with 82% of respondents choosing between 8 and 12 characteristics that ‘matter most’ to them and 92% of respondents provided an importance score for between 5 and 9 of those characteristics. The distribution of number of characteristics that farmers provided an importance score for was also highly concentrated at 20 characteristics, but five respondents chose to score fewer than the possible 20.

11 The farmer’s scale was defined as 1 = ‘Not at all important’, 2 = ‘Low importance’, 3 = ‘Moderately important’ and 4 = ‘Most important’. The farmer’s scale was mapped onto the public’s scale so that the farmer’s new scale was defined as 1 = ‘Not at all important’, 3 = ‘Slightly important’, 5 = ‘Moderately important’ and 7 = ‘Extremely important’.

12 Groundswell NZ is a ‘grassroots volunteer-driven advocacy group seeking a halt to, and rewrite of, unworkable regulations which unfairly impact farmers and rural communities.’ https://www.groundswellnz.co.nz/our-mission.
