# Supplementary material for: Perceptions of the ‘good farmer’ and social licence to operate in Aotearoa New Zealand
Source: J R Soc N Z. 2024 May 26;55(6):1589–609. doi: 10.1080/03036758.2024.2351910 (PMC12315156; doi:10.1080/03036758.2024.2351910)
Supplement: Supplemental material [file TNZR_A_2351910_SM9138.docx]

Supplementary material

Table SM1a. Multi-linear regression results comparing public and farmer perceptions of importance of ‘good farming’ characteristics (1 to 7).

|  | (1) | (2) | (3) | (4) | (5) | (6) | (7) |
| --- | --- | --- | --- | --- | --- | --- | --- |
| Independent variables | Uses Regional council or industry-developed GMPs | Has strong place attachment/ connection to a farm or place | Follows government rules/ regulations | Produces high-value end products | Has strong consideration of the Mauri of a place | Plants trees throughout their farm | Controls pests |
| Difference between public and farmer perceptions of importance | 0.82** | 0.67* | 0.57 | 0.35 | 0.30 | 0.12 | -0.15 |
|  | (0.37) | (0.37) | (0.35) | (0.38) | (0.37) | (0.33) | (0.24) |
| Above average age (=1) | 0.60*** | 0.0022 | 0.31*** | 0.28* | 0.28 | 0.57*** | 0.22** |
|  | (0.11) | (0.095) | (0.072) | (0.15) | (0.22) | (0.14) | (0.11) |
| Female (=1) | 0.61*** | 0.34*** | 0.27*** | -0.10 | 0.15 | 0.0041 | 0.12 |
|  | (0.12) | (0.096) | (0.079) | (0.15) | (0.22) | (0.14) | (0.10) |
| Māori (=1) | -0.063 | -0.24** | -0.041 | -0.27 | 0.093 | -0.19 | -0.23* |
|  | (0.14) | (0.12) | (0.11) | (0.17) | (0.27) | (0.18) | (0.12) |
| NZ European (=1) | 0.31** | 0.10 | 0.28*** | 0.015 | 0.11 | 0.13 | -0.0042 |
|  | (0.14) | (0.11) | (0.10) | (0.17) | (0.24) | (0.17) | (0.11) |
| Constant | 3.98*** | 4.94*** | 5.18*** | 5.09*** | 4.70*** | 4.87*** | 5.51*** |
|  | (0.40) | (0.39) | (0.39) | (0.44) | (0.48) | (0.37) | (0.27) |
| Observation | 511 | 684 | 769 | 290 | 200 | 382 | 591 |
| *R*-squared | 0.121 | 0.047 | 0.055 | 0.028 | 0.012 | 0.054 | 0.020 |
| *F*-stat | 13.6 | 5.22 | 8.14 | 1.72 | 0.51 | 5.01 | 2.28 |
| Average public score | 5.54 | 5.83 | 6.24 | 5.45 | 5.28 | 5.29 | 5.44 |
| Average farmer score | 4.72 | 5.15 | 5.67 | 5.10 | 4.98 | 5.17 | 5.58 |

Notes: Dependent variable was level of importance of ‘good farmer’ characteristic on a 7-point Likert scale where 1 = ‘Not at all important’, 2 = ‘Low importance’, 3 = ‘Sightly important’, 4 = ‘Neutral, 5 = ‘Moderately important’, 6 = ‘Very important’ and 7 = ‘Extremely important’. Each characteristic was analysed separately. Standard errors are in parentheses below regression coefficients. Difference is defined as average difference of public scores from the farmer scores. Stars represent significance where * is p<.10, ** is p<.05 and *** is p<.01. Regression was estimated using OLS with robust standard errors.

Table SM1b. Multi-linear regression results comparing public and farmer perceptions of importance of ‘good farming’ characteristics (8 to 14).

|  | (8) | (9) | (10) | (11) | (12) | (13) | (14) |
| --- | --- | --- | --- | --- | --- | --- | --- |
| Independent variables | Reduces chemical inputs where possible | Contributes to the local community | Has and follows a farm management plan | Uses a whole landscape approach/ ki uta ki tai | Is profitable | Provides public transparency around farming practices | Enhances native biodiversity on their farm |
| Difference between public and farmer perceptions of importance | -0.37 | -0.43 | -0.52 | -0.61* | -0.67** | -0.75** | -0.75** |
|  | (0.29) | (0.32) | (0.38) | (0.35) | (0.27) | (0.29) | (0.30) |
| Above average age (=1) | 0.31*** | 0.38** | 0.82*** | -0.12 | 0.24 | 0.26** | 0.17 |
|  | (0.11) | (0.19) | (0.30) | (0.26) | (0.16) | (0.12) | (0.17) |
| Female (=1) | 0.085 | 0.061 | -0.0035 | -0.063 | -0.026 | 0.28** | 0.11 |
|  | (0.11) | (0.18) | (0.28) | (0.24) | (0.15) | (0.12) | (0.16) |
| Māori (=1) | 0.0023 | 0.76*** | 0.054 | -0.66** | -0.20 | -0.35** | 0.073 |
|  | (0.13) | (0.21) | (0.31) | (0.28) | (0.18) | (0.15) | (0.17) |
| NZ European (=1) | 0.21 | 0.59*** | 0.10 | 0.24 | 0.069 | -0.36*** | -0.28 |
|  | (0.13) | (0.20) | (0.30) | (0.24) | (0.17) | (0.13) | (0.18) |
| Constant | 5.77*** | 4.67*** | 4.98*** | 5.76*** | 5.86*** | 5.96*** | 5.92*** |
|  | (0.31) | (0.41) | (0.47) | (0.45) | (0.34) | (0.33) | (0.37) |
| Observation | 486 | 210 | 130 | 169 | 263 | 452 | 275 |
| *R*-squared | 0.034 | 0.103 | 0.102 | 0.077 | 0.057 | 0.058 | 0.038 |
| *F*-stat | 3.11 | 4.75 | 3.51 | 2.38 | 2.78 | 4.84 | 2.02 |
| Average public score | 5.74 | 5.07 | 4.81 | 5.03 | 5.26 | 5.12 | 5.13 |
| Average farmer score | 6.11 | 5.50 | 5.33 | 5.65 | 5.93 | 5.87 | 5.88 |

Notes: Dependent variable was level of importance of ‘good farmer’ characteristic on a 7-point Likert scale where 1 = ‘Not at all important’, 2 = ‘Low importance’, 3 = ‘Sightly important’, 4 = ‘Neutral, 5 = ‘Moderately important’, 6 = ‘Very important’ and 7 = ‘Extremely important’. Each characteristic was analysed separately. Standard errors are in parentheses below regression coefficients. Difference is defined as average difference of public scores from the farmer scores. Stars represent significance where * is p<.10, ** is p<.05 and *** is p<.01. Regression was estimated using OLS with robust standard errors.

Table SM1c. Multi-linear regression results comparing public and farmer perceptions of importance of ‘good farming’ characteristics (15 to 20).

|  | (15) | (16) | (17) | (18) | (19) | (20) |
| --- | --- | --- | --- | --- | --- | --- |
| Independent variables | Complies with biosecurity requirements | Uses overwintering practices that focus on animal welfare | Looks after their farm staff in an equitable way | Manages the farm in an ethical way | Cares for the welfare of their stock | Manages the farm in an environmentally friendly way |
| Difference between public and farmer perceptions of importance | -0.90*** | -0.93*** | -0.94*** | -0.95*** | -0.97*** | -1.02*** |
|  | (0.27) | (0.33) | (0.13) | (0.18) | (0.12) | (0.23) |
| Above average age (=1) | 0.39*** | 0.67*** | 0.24*** | 0.14 | 0.24** | 0.44*** |
|  | (0.11) | (0.24) | (0.083) | (0.12) | (0.10) | (0.12) |
| Female (=1) | 0.57*** | 0.62*** | 0.34*** | 0.38*** | 0.30*** | -0.033 |
|  | (0.11) | (0.22) | (0.086) | (0.12) | (0.10) | (0.11) |
| Māori (=1) | -0.011 | 0.25 | -0.38*** | -0.067 | -0.32*** | -0.051 |
|  | (0.13) | (0.23) | (0.11) | (0.15) | (0.12) | (0.14) |
| NZ European (=1) | 0.28** | 0.12 | 0.20* | 0.087 | 0.078 | 0.014 |
|  | (0.13) | (0.26) | (0.10) | (0.13) | (0.12) | (0.14) |
| Constant | 5.72*** | 5.49*** | 6.44*** | 6.27*** | 6.56*** | 6.28*** |
|  | (0.32) | (0.45) | (0.17) | (0.23) | (0.17) | (0.28) |
| Observation | 471 | 139 | 713 | 443 | 472 | 502 |
| *R*-squared | 0.118 | 0.179 | 0.097 | 0.061 | 0.095 | 0.065 |
| *F*-stat | 12 | 7.72 | 21.8 | 8.44 | 19.4 | 9.62 |
| Average public score | 5.47 | 5.30 | 5.82 | 5.62 | 5.82 | 5.42 |
| Average farmer score | 6.37 | 6.23 | 6.76 | 6.57 | 6.79 | 6.44 |

Notes: Dependent variable was level of importance of ‘good farmer’ characteristics on a 7-point Likert scale where 1 = ‘Not at all important’, 2 = ‘Low importance’, 3 = ‘Sightly important’, 4 = ‘Neutral, 5 = ‘Moderately important’, 6 = ‘Very important’ and 7 = ‘Extremely important’. Each characteristic was analysed separately. Standard errors are in parentheses below regression coefficients. Difference is defined as average difference of public scores from the farmer scores. Stars represent significance where * is p<.10, ** is p<.05 and *** is p<.01. Regression was estimated using OLS with robust standard errors.

Table SM2. Multi-linear regression results comparing average demographics of respondents to the public survey by the number of ‘good farming’ characteristics where perceptions align between the public and farmers.

|  | | (1) | (2) | (3) | (4) | (5) | (6) | (7) | (8) |
| --- | --- | --- | --- | --- | --- | --- | --- | --- | --- |
| Independent variables | | Age group | Female | Māori | NZ European | Age group | Female | Māori | NZ European |
| Number of ‘good farmer ‘characteristics that aligned between the public and farmers: | At least 1 | 0.49*** | 0.11* | -0.12** | 0.15*** |  |  |  |  |
|  |  | (0.12) | (0.054) | (0.054) | (0.055) |  |  |  |  |
|  | 1 |  |  |  |  | 0.12 | 0.0026 | -0.058 | 0.072 |
|  |  |  |  |  |  | (0.16) | (0.067) | (0.066) | (0.067) |
|  | 2 |  |  |  |  | 0.34** | 0.10* | -0.096 | 0.16*** |
|  |  |  |  |  |  | (0.15) | (0.061) | (0.060) | (0.061) |
|  | 3 |  |  |  |  | 0.63*** | 0.13** | -0.11* | 0.17*** |
|  |  |  |  |  |  | (0.15) | (0.061) | (0.059) | (0.061) |
|  | 4 |  |  |  |  | 0.50*** | 0.17*** | -0.18*** | 0.16** |
|  |  |  |  |  |  | (0.16) | (0.063) | (0.060) | (0.063) |
|  | 5 |  |  |  |  | 0.83*** | 0.14** | -0.21*** | 0.18*** |
|  |  |  |  |  |  | (0.19) | (0.070) | (0.063) | (0.068) |
|  | 6 |  |  |  |  | 0.39 | 0.12 | -0.16* | 0.060 |
|  |  |  |  |  |  | (0.27) | (0.10) | (0.090) | (0.100) |
|  | 7 |  |  |  |  | 1.27*** | 0.062 | -0.060 | 0.14 |
|  |  |  |  |  |  | (0.43) | (0.14) | (0.13) | (0.13) |
| Constant | | 2.93*** | 0.40*** | 0.39*** | 0.53*** | 2.93*** | 0.40*** | 0.39*** | 0.53*** |
|  |  | (0.11) | (0.052) | (0.052) | (0.053) | (0.11) | (0.052) | (0.052) | (0.053) |
| Observations | | 1,084 | 1,084 | 1,084 | 1,084 | 1,084 | 1,084 | 1,084 | 1,084 |
| *R*-squared | | 0.007 | 0.004 | 0.006 | 0.008 | 0.026 | 0.013 | 0.017 | 0.014 |

Notes: Dependent variable is demographic characteristic where ‘age groups’ are on a seven-point scale and ‘female’, ‘Māori’, and ‘NZ European’ are binary equally 1 if respondent self-identified as those characteristics. ‘Age groups’ are defined as 1 if respondent is <20 years old, 2 if respondents is 21 to 30 years old, 3 if respondent is 31 to 40 years old, 4 if respondents is 41 to 50 years old, 5 if respondent 51 to 60 years old, 6 if respondent is 61 to 70 years old, and 7 if respondent is 71 years or older. Each demographic was analysed separately. Standard errors are in parentheses below regression coefficients. Base group in models is '0 characteristics align'. Stars represent significance where * is p<.10, ** is p<.05 and *** is p<.01. Regression was estimated using OLS with robust standard errors. No difference in estimates for models (2) to (4) and (6) to (8) if run using maximum likelihood instead of OLS.

Table SM3. Multi-linear regression results of public perceptions of trustworthiness of farmers as a source of information on farming by number of ‘good farming’ characteristics where perceptions align between the public and farmers.

|  | Independent variables | (1) | (2) | (3) |
| --- | --- | --- | --- | --- |
| Number of ‘good farmer’ characteristics that aligned between the public and farmers: | 1 | 0.16 | 0.16 | 0.16 |
|  |  | (0.19) | (0.19) | (0.19) |
|  | 2 | 0.59*** | 0.59*** | 0.59*** |
|  |  | (0.17) | (0.17) | (0.17) |
|  | 3 | 0.57*** | 0.57*** | 0.56*** |
|  |  | (0.17) | (0.17) | (0.17) |
|  | 4 | 0.69*** | 0.69*** | 0.69*** |
|  |  | (0.17) | (0.17) | (0.17) |
|  | 5 | 0.68*** | 0.68*** | 0.68*** |
|  |  | (0.19) | (0.19) | (0.19) |
|  | 6 | 0.42 | 0.42 | 0.42 |
|  |  | (0.29) | (0.29) | (0.29) |
|  | 7 | 0.33 | 0.33 | 0.34 |
|  |  | (0.39) | (0.39) | (0.39) |
|  | Above average age (=1) | 0.047 | 0.048 | 0.046 |
|  |  | (0.084) | (0.084) | (0.084) |
|  | Female (=1) | 0.41*** | 0.42*** | 0.41*** |
|  |  | (0.082) | (0.082) | (0.082) |
|  | Māori (=1) | -0.16 | -0.16* | -0.16 |
|  |  | (0.097) | (0.098) | (0.097) |
|  | NZ European (=1) | 0.014 | 0.016 | 0.012 |
|  |  | (0.087) | (0.087) | (0.088) |
|  | Familiar with primary industries (=1) |  | 0.19 |  |
|  |  |  | (0.22) |  |
|  | Live in urban centre (=1) |  |  | -0.053 |
|  |  |  |  | (0.091) |
|  | Live in rural area (=1) |  |  | -0.030 |
|  |  |  |  | (0.15) |
|  | Constant | 5.02*** | 4.83*** | 5.06*** |
|  |  | (0.16) | (0.27) | (0.18) |
|  | Observation | 1,084 | 1,084 | 1,084 |
|  | *R*-squared | 0.065 | 0.066 | 0.065 |
|  | F-stat | 7.19 | 6.69 | 6.09 |

Notes: Dependent variable is perceived trustworthiness of farmers as a source of information on a 7-point Likert scale where 1 = ‘Completely untrustworthy, 2 = ‘Mostly untrustworthy, 3 = ‘Somewhat untrustworthy, 4 = ‘Neutral, 5 = ‘Somewhat trustworthy’, 6 = ‘Mostly trustworthy’, and 7 = ‘Completely trustworthy. Standard errors are in parentheses below regression coefficients. Estimate for each number of ‘good farmer’ characteristic that align is the difference in trustworthy scores between public respondents who have that number of characters align from public and respondents who did not have any characteristics align. ‘Familiarity with primary industries’ is defined as 1 if respondent self-reported that they were familiar with at least one primary industry where ‘Familiarity’ is defined as having visited, worked on or have knowledge of a primary industry (e.g. sheep and beef, dairying). Stars represent significance where * is p<.10, ** is p<.05 and *** is p<.01. Regression is estimated using OLS with robust standard errors.

Table SM4. Multi-linear regression results comparing public and farmer perceptions of trustworthiness of farmers as a source of information by number of ‘good farming’ characteristics where perceptions align between the public and farmers.

|  | (1) | (2) | (3) | (4) | (5) | (6) | (7) | (8) |
| --- | --- | --- | --- | --- | --- | --- | --- | --- |
|  | Number of ‘good farmer’ characteristics that aligned between the public and farmers | | | | | | | |
| Independent variables | 0 | 1 | 2 | 3 | 4 | 5 | 6 | 7 |
| Difference between public and farmer perceptions of trustworthiness | -0.085 | 0.51** | 0.55** | 0.54** | 0.58** | 0.54** | 0.27 | -0.0017 |
|  | (0.33) | (0.22) | (0.22) | (0.22) | (0.23) | (0.24) | (0.33) | (0.48) |
| Above average age (=1) | 0.069 | 0.065 | 0.039 | -0.048 | -0.13 | -0.22 | -0.43 | -0.27 |
|  | (0.27) | (0.086) | (0.090) | (0.10) | (0.13) | (0.19) | (0.34) | (0.40) |
| Female (=1) | 0.14 | 0.48*** | 0.40*** | 0.36*** | 0.30** | 0.38* | 0.082 | 0.044 |
|  | (0.29) | (0.083) | (0.088) | (0.10) | (0.13) | (0.19) | (0.35) | (0.49) |
| Māori (=1) | 0.16 | -0.22** | -0.28*** | -0.33** | -0.34* | -0.25 | -0.097 | 0.78 |
|  | (0.29) | (0.10) | (0.11) | (0.13) | (0.18) | (0.27) | (0.44) | (0.54) |
| NZ European (=1) | 0.29 | -0.0098 | -0.078 | -0.048 | 0.085 | 0.24 | 0.29 | 0.073 |
|  | (0.31) | (0.091) | (0.096) | (0.11) | (0.15) | (0.22) | (0.38) | (0.44) |
| Constant | 4.94*** | 5.05*** | 5.18*** | 5.24*** | 5.22*** | 5.09*** | 5.36*** | 5.37*** |
|  | (0.43) | (0.25) | (0.25) | (0.26) | (0.28) | (0.36) | (0.62) | (0.71) |
| Observation | 109 | 1,015 | 875 | 626 | 375 | 185 | 69 | 35 |
| *R*-squared | 0.020 | 0.042 | 0.039 | 0.043 | 0.047 | 0.066 | 0.053 | 0.078 |
| *F*-stat | 0.53 | 8.33 | 6.64 | 4.88 | 3.21 | 2.33 | 0.82 | 0.69 |
| Average public score | 5.17 | 5.77 | 5.84 | 5.85 | 5.89 | 5.84 | 5.61 | 5.43 |
| Average farmer score | 5.25 | 5.26 | 5.29 | 5.31 | 5.32 | 5.30 | 5.35 | 5.43 |

Notes: Dependent variable is perceived trustworthiness of farmers as a source of information on a 7-point Likert scale where 1 = ‘Completely untrustworthy, 2 = ‘Mostly untrustworthy, 3 = ‘Somewhat untrustworthy, 4 = ‘Neutral, 5 = ‘Somewhat trustworthy’, 6 = ‘Mostly trustworthy’, and 7 = ‘Completely trustworthy. Each alignment level is analysed separately. Standard errors are in parentheses below regression coefficients. Difference is defined as average difference of public scores from the farmer scores. Stars represent significance where * is p<.10, ** is p<.05 and *** is p<.01. Regression is estimated using robust standard errors.
